# Abdominal angiostrongyliasis, report of two cases and analysis of published reports from Colombia

**DOI:** 10.7705/biomedica.5043

**Published:** 2020-06-30

**Authors:** Fernando Bolaños, Leonardo F. Jurado, Rina L. Luna-Tavera, Jaime M. Jiménez

**Affiliations:** 1 Departamento de Patología, Hospital Universitario Hernando Moncaleano Perdomo, Neiva, Colombia Departamento de Patología Hospital Universitario Hernando Moncaleano Perdomo Neiva Colombia; 2 Departamento de Patología, Hospital Universitario Departamental de Nariño, Pasto, Colombia Departamento de Patología Hospital Universitario Departamental de Nariño PastoColombia; 3 Departamento de Patología y Laboratorios, Hospital Universitario Fundación Santa Fe de Bogotá, Bogotá, D.C., Colombia Departamento de Patología y Laboratorios Hospital Universitario Fundación Santa Fe de Bogotá Bogotá, D.C Colombia; 4 Departamento de Microbiología, Facultad de Medicina, Universidad Nacional de Colombia, Bogotá, D.C., Colombia Universidad Nacional de Colombia Departamento de Microbiología Facultad de Medicina, Universidad Nacional de Colombia Bogotá, D.C Colombia; 5 Facultad de Medicina, Fundación Universitaria Sanitas, Bogotá, D.C., Colombia Facultad de Medicina, Fundación Universitaria Sanitas Bogotá, D.C Colombia

**Keywords:** Angiostrongylus, *Strongylida* infections/diagnosis, case reports, Colombia, Angiostrongylus, infecciones por *Strongylida*/diagnóstico, informes de casos, Colombia

## Abstract

Abdominal angiostrongyliasis is a parasitic zoonosis, endemic in the American continent. Its etiological agent is *Angiostrongylus costaricensis*, a nematode whose definitive hosts are rats and other rodents and the intermediate hosts, slugs. Mammals acquire the infection by consuming vegetables contaminated with L3 larvae. The disease shows a heterogeneous clinical spectrum and given its low incidence its diagnosis is a great challenge.

In Colombia, the first case was reported in 1979 and until 1998, only five additional cases have been reported. However, in the last two decades, no new cases were reported. Here we discuss two cases of children from Huila and Caquetá departments who developed the disease. Both cases required long in-patient care and multiple surgical interventions. The diagnosis was achieved by histopathological observation of parasitic elements inside the mesenteric arteries. One of the children died while the other fully recovered.

We discuss the epidemiology, pathogenic cycle, clinical presentation, diagnosis, and prevention strategies of this disease paying particular attention to our patients’ features and the Colombian context.

## Resumen

La angiostrongiloidiasis abdominal es una zoonosis parasitaria endémica en el continente americano. Su agente etiológico es el nematodo *Angiostrongylus costaricensis,* cuyos huéspedes definitivos son los roedores y, los intermediarios, los caracoles y las babosas, por lo que se adquiere al consumir vegetales contaminados con larvas en estadio 3. La presentación clínica es muy variada y, dada su rareza, su diagnóstico es un desafío.

En Colombia el primer caso se reportó en 1979 y, desde entonces hasta 1998, se han informado cinco casos más, aunque en las últimas dos décadas no se había reportado ningún caso. Se describen aquí dos casos de angiostrongiloidiasis en niños provenientes de los departamentos de Huila y Caquetá que requirieron una larga hospitalización y múltiples intervenciones quirúrgicas. El diagnóstico se logró al observar en los especímenes quirúrgicos larvas and huevos cuya morfología sugería una infección por nematodos; uno de los pacientes murió y el otro se recuperó satisfactoriamente.

Se discuten la epidemiología, la patogenia, la presentación clínica, el diagnóstico y las estrategias de prevención de esta parasitosis, con énfasis en las características particulares de los casos descritos y en el contexto colombiano.

Human angiostrongyliasis is a parasitic and zoonotic disease caused by the nematode species *Angiostrongylus costaricensis* and *A. cantonensis*, which belong to the Metastrongylidae family (Leiper, 1908) ^1^. *Angiostrongylus costaricensis*, which produces abdominal disease, was described by Morera and Céspedes in Costa Rica in 1952 ^2^^,^^3^.

*Angiostrongylus cantonensis,* which causes eosinophilic meningoencephalitis, was reported infecting the pulmonary arteries and hearts of domestic rats in Guangzhou (Canton), China, by Chen in 1935 ^4^. In both cases, rats are the main definitive hosts and snails are the intermediate hosts ^1^^,^^3^.

To date, 18 species of *Angiostrongylus* have been identified around the world ^5^. Their taxonomic classification relies on the morphological characteristics of the rays of the copulatory bursa, its host group specificity, and the organ where the adult worms are located while infecting the host ^5^^,^^6^.

*Angiostrongylus costaricensis*, endemic in South America and the Caribbean, is responsible for a pathologic abdominal syndrome characterized by the presence of the nematode and its eggs inside the mesenteric arteries ^2^. Histologically it is characterized by eosinophilic infiltration, vascular abnormality, and granulomatous reaction ^7^. Morera and Céspedes in Costa Rica first reported this condition in 1952, and in 1971 they described the parasite using surgical specimens obtained from a patient who had an appendicitis-like clinical syndrome ^8^. The current natural distribution of this parasite ranges from the southern United States to northern Argentina ^9^ and human cases have been reported in Brazil, Colombia, Costa Rica, Dominican Republic, El Salvador, France, Guadalupe, Honduras, Martinique, México, Nicaragua, Panamá, Puerto Rico, Spain, the United States, Venezuela, and Zaire ^10^^,^^11^.

The adult nematode has a filiform morphology and its cephalic pole is round. The caudal end is knoblike and its esophagus is club-shaped. Its copulatory bursa is slightly asymmetric and well developed. The dorsal ray is short and bifurcates in branches ending in sharp tips ^3^^,^^5^^,^^6^.

**Figure 1:**
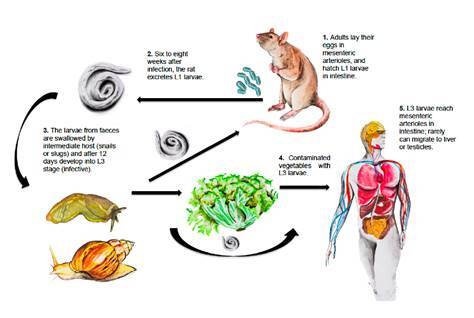
*Angiostrongylus costaricensis* life cycle Rats and other rodents are definitive hosts. Humans get infected when third-stage larvae are ingested. In rats, adult female worms produce approximately 15,000 eggs daily. Eggs are carried to the mesenteric arterioles and break into the intestinal lumen where they hatch. First-stage larvae (L1) are excreted with the feces and then they are swallowed by intermediate hosts (snails or slugs) and develop into third-stage larvae (L3) (infective). Human beings occasionally acquire the infection by eating snails, slugs or contaminated vegetables containing the infective larvae. The larvae enter the bloodstream in the intestine wall and can move to the liver or other abdominal organs causing angiostrongyliasis. Illustrations by Óscar Chávez, photography by David Bolaños, design by Leonardo F. Jurado

The most important definitive hosts are rodent species, the main being *Sigmodon hispidus* (hispid cotton rat) ^1^^,^^11^. Other species such as *Rattus rattus*, *Zygodontomys microtinus*, *Orizomys fulvesceus*, and *Orizomys caliginosus* are also relevant ^12^. In some cases, even domestic dogs can be reservoirs ^13^. Intermediate hosts are slugs of the Veronicellidae family such as *Sarasinula plebeia* and snails such as *Lissachatina fulica*, but several other mollusks may also play a role as intermediate hosts ^14^^-^^17^. Adult worms reside inside the mesenteric arteries of rodents where females lay their eggs ^18^. Humans are accidental hosts due to the ingestion of contaminated vegetables, raw or undercooked snails, and mollusks containing L3 larvae are necessary to become infected ^19^^-^^22^. The cycle is not completed when host-parasite adaptation fails ^23^ (figure 1).

In humans, both the eggs and the larvae provoke a severe inflammatory reaction in the ileocecal mucosa characterized by a granulomatous response with marked eosinophilic infiltrates. These pathological phenomena can trigger intestinal bleeding, perforation, and/or intestinal stenosis with subsequent obstruction ^24^^,^^25^.

In epidemiological terms, incidence or prevalence data are scarce. In Brazil, Graeff-Teixeira, *et al.* reported that seroprevalence in the general population was 28% ^26^. The clinical presentation is varied and ranges from asymptomatic to severe manifestations that require emergency surgery ^27^.

We describe here two cases of abdominal angiostrongyliasis in two children from the south-central region of the country (Huila and Caquetá departments). Interestingly, our cases are the first ones to be reported in Colombia in this century. The last case was reported in 1998 ^28^.

We discuss the epidemiology, pathogenic course, clinical presentation, and diagnosis of the cases focusing on their specific features. We also analyzed the previous cases reported in Colombia.

The report was approved by the Medical Ethics Committee in Neiva’s *Hospital Universitario Hernando Moncaleano Perdomo*.

## Case 1

A twelve-year-old boy from Florencia (Caquetá) previously diagnosed with Down’s syndrome was received in the emergency room referred from another hospital due to abdominal sepsis. In the referring hospital, he had already received multiple antibiotic schemes.

The patient had consulted 35 days before in Florencia with diarrhea and systemic inflammatory signs. Blood tests showed a high white blood cell count, neutrophilia, and positive acute phase reactants. An abdominal ultrasound evidenced cholelithiasis and free peritoneal fluid while the computed tomography (CT) scan showed hepatomegaly and nephrolithiasis.

At admission, the patient was hemodynamically stable, with mucocutaneous pallor and a body temperature of 36.8 °C. The chest physical examination was normal, the abdomen was found distended (80 cm in perimeter), and palpation was painful over the lower right quadrant, but no peritoneal irritation signs were found. The white blood cell count was 38,200 per mm3 (90.5%, neutrophils; 4%, lymphocytes; 4%, monocytes, and 0.4%, eosinophils); hemoglobin level, 9.3 g/dl, and C reactive protein, 31 mg/l.

The abdominal CT scan was repeated and it showed severe intestinal distention affecting the jejunum and ileum, a mass located in the right iliac fossa, and free liquid in the cavity. The bone marrow aspirate was normal and only megakaryocyte and plasmocyte counts had increased.

A laparotomy was performed given the patient’s clinical and radiological findings, which revealed generalized peritonitis and a plastron affecting the distal ileum, the greater omentum, and the appendix. The distal ileum was widely necrotic and the jejunum and ileum were severely distended. Ascitic fluid was drained and 5,300 ml of purulent fluid were collected, the plastron was liberated, and the appendix, part of the omentum and a portion of ileum were resected.

**Figure 2:**
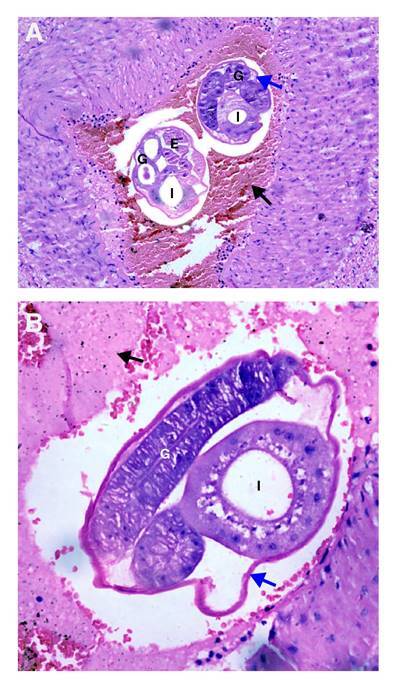
A and B. Mesenteric small artery with a thrombus (black arrow). Adult female worms cross-sectioned at different levels are observed inside. Note the cuticle (blue arrow), multinucleated intestine (I), and well-developed gonads (G) with eggs inside (E). Hematoxylin and eosin, 40X.

Between day two and day 22 after the surgery, the percentage of eosinophils progressively increased reaching 58.9% on day 29. A histological study of the surgical specimens showed intestinal ischemic necrosis due to vascular obstruction by larvae and eggs whose morphology suggested a nematode infection. The blood vessels of mesenteries, appendix, and lymphatic nodes also showed eggs. The morphological features and histological pattern allowed for the identification of the nematode as *A. costaricensis* (figure 2 ).

During the subsequent weeks, the patient showed a poor clinical response and required four additional surgical interventions and eleven peritoneal lavages. Other intestinal and mesenteric segments were resected because of perforation, necrosis, and adhesions.

The clinical course was unfavorable with significant weight loss. Gram- negative bacteria were isolated from both blood and peritoneal cultures. The patient received various antibiotic schemes and also IgM-enriched intravenous immunoglobulin, but he passed away after 97 days of his admission.

## Case 2

A four-year-old boy from the rural area of Pitalito municipality (Huila) was admitted to the emergency room after four days of pain in the lower right quadrant, fever, and dysuria. An intestinal mass was incidentally found during the appendectomy that was practiced given his acute abdominal signs. The appendix was normal, but an intraluminal mass with surrounding lymphatic nodules was found in the cecum. The mass had adhered to the anterior abdominal wall. The surgeon resected the appendix, but not the mass, and he referred the patient to a bigger hospital in Neiva.

When admitted in Neiva, he was in good general condition, he had no fever, and his vital signs were within the normal ranges. The abdomen was soft, depressible, and showed no palpable masses; there was peristalsis and the surgical wound was normal.

An initial blood test showed a white blood cell count of 9,200 per mm3 (58.4%, neutrophils; 22.5%, lymphocytes; 4.2%, monocytes, and 12.8%, eosinophils). The hemoglobin level was 10.6 g/l and reactive C protein was 5.7 mg/l. Considering his clinical and laboratory findings, metronidazole and amikacin were started.

An abdominal CT scan showed concentric and circumferential wall thickening involving the cecum, the ascending colon, and the right colic flexure. The lesion also affected the surrounding fat tissue and many lymphatic nodules were also observed.

Examination by the pediatric surgeon did not evidence acute abdomen signs or abdominal masses. The patient continued to show good clinical evolution. On the fifth day after admission, another CT scan was performed. It showed a mass occupying the low right quadrant, which suggested a Burkitt lymphoma. An exploratory laparotomy was programmed.

The histological study of the appendix resected during the first intervention in Pitalito showed lymphoid hyperplasia. After 12 days of admission, a blood test revealed a white blood cell count of 10,600 per mm3 (10.9% eosinophils). The laparotomy was performed on day 13 and the main findings were a plastron located in the lower right quadrant involving the omentum, the distal ileum, and the cecum, as well as multiple adhesions and a significant affectation of the jejunal lumen. Macroscopically, the cecum and the colon lesions showed a neoplastic appearance. Therefore, a resection from the distal ileum to the transverse colon was carried out.

**Figure 3:**
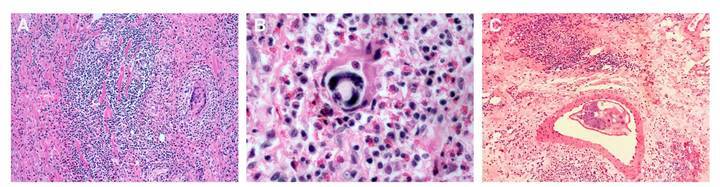
A. Colonic submucosa surrounded by intense eosinophilic inflammation and granulomatous reaction with giant multinucleated cells. B. Intestinal tissue surrounded by severe eosinophilic infiltration and a developing larva. C. Mesenteric tissue affected by granulomatous eosinophilic reaction; note a small artery containing an adult female worm. Hematoxylin and eosin, 40X.

After this, the histological study showed ulcerated intestinal mucosa, thromboembolism in arterioles, eosinophilic vasculitis, chronic necrotizing granulomatous inflammation involving the whole intestinal wall, abundant lymphoplasmacytoid and histiocytoid infiltrates, and eosinophilic clusters. Inside granulomas, giant multinucleated cells, and a high number of eggs were identified, and several larval structures inside the intestinal arterioles were evidenced. The morphological features and the histological pattern observed allowed their identification as *A. costaricensis* (figure 3).

Two ivermectin plus albendazole doses were administered for five days. The clinical evolution was satisfactory and after twenty days the patient was discharged.

### Previous cases reported in Colombia

We analyzed the previous cases in Colombia reported in the literature after a search using the MesH terms “angiostrongyliasis”, “*Angiostrongylus costaricensis”*, and “Colombia” in Medline (PubMed), SciELO, Google, and Google Scholar. Interestingly, we found that since 1998 no cases had been reported in Colombia ^28^.

## Discussion

Morera and Céspedes first described abdominal angiostrongyliasis in Costa Rican patients in 1952 ^2^, and twenty years later the etiological agent was identified ^3^. Costa Rica is the most endemic country in the region as almost 90% of the cases reported worldwide originate there ^11^. This disease is more frequent in children and males as has been evident in Colombia where five out of the eight cases reported to date were in children less than 12-year-old while six were in males ^28^.

Human beings are accidental hosts and infection occurs after ingestion of mollusks, snails or contaminated vegetables containing the L3 larvae ^19^^,^^22^. It has been informed that its frequency increases during rainy months because this facilitates the reproduction of the mollusks ^29^. In fact, both of our cases were diagnosed during the rainy season (March).

The real incubation period is unknown, but it is estimated to be three to four weeks. The eggs and larvae induce an intense inflammatory response generating a granulomatous reaction with eosinophilia that may evolve to intestinal stenosis.

Pathological studies have revealed that the most frequently affected organs are the appendix, the ileum, the colon, and the surrounding lymph nodes ^29^. In a case series where 90 patients were surgically intervened, 36 had only one organ affected (colon, ileum or appendix), and in 25 there was the involvement of at least three segments (colon, appendix, and cecum). Only in two cases were the cecum and sigmoid affected while one case had disseminated disease ^30^. In this series, only three cases showed affectation of just one organ (small intestine and appendix) while the remaining five cases presented affectation of at least two organs; in our cases, we identified omentum and jejunal involvement. Cases of extraintestinal invasion are very rare, but liver and testicles can be affected. Some authors suggest that anthelmintic therapy stimulates worm migration, which increases the risk of extraintestinal disease ^25^.

Macroscopically, two patterns are the most frequent: one characterized by the thickening of the intestinal wall (pseudoneoplastic) and the other showing a congestive necrotic pattern (ischemic-congestive) ^11^^,^^28^. In the two cases we described here, the 12-year-old patient had a congestive necrotic pattern while the four-year-old boy developed the pseudoneoplastic pattern. Additionally, hepatomegaly was identified in one of our patients, an alteration described in 50% of cases. In a case reported in Colombia in 1987 ^28^, the authors described severe vascular involvement causing arterial rupture, i.e., the hemorrhagic form of the disease, which is frequently fatal.

Histologically, two predominant patterns are usually described: severe eosinophilic infiltration involving all layers of the intestinal wall and a granulomatous reaction and eosinophilic vasculitis affecting capillaries, arteries, veins, and even lymphatic vessels ^24^. Both patients described here developed granulomatous inflammation with abundant eosinophil infiltrates (table 1).

**Table 1.** General characteristics of the cases described

Sex	Age	Origin	Clinical presentation	Organ affected	Pathological findings	Outcome
Male	12	Florencia (Caqueta)	Diarrhea, systemic inflammatory signs, abdominal distension, painful palpation in low - right quadrant. WBC count: 38,200 per mm3, 0.4% eosinophils	Distal ileum, omentum, and appendix	Hepatomegaly.Ischemic necrosis and granulomatous inflammation involving distal ileum, omentum, mesentery, and lymphatic nodes. Larvae and eggs inside vessels from all affected organs	Death
Male	4	Pitalito (Huila)	Acute appendicitislike case. WBC count: 10,600 per mm3, 10.9% eosinophils	Jejunum, distal ileum, cecum, appendix, and omentum	Granulomatous inflammation and eosinophilic vasculitis involving jejunum, distal ileum, and cecum. Larvae and eggs over the affected tissue and parasites inside the vessels	Full recovery

WBC: White blood cells

 Regarding the clinical presentation, general symptoms such as malaise and myalgias are usually present in all cases ^11^. Frequently, clinical findings mimic those of appendicitis ^11^^,^^30^. A systematic review of case reports informed that abdominal pain was present in 84% of cases, vomiting in 50%, diarrhea in 28%, and constipation in 14.2% ^11^. Generally, abdominal pain is localized in the right lower quadrant. Sometimes a painful mass or plastron can also be found in there, which is usually interpreted as complicated apendicitis or neoplastic lesion. In a case series of 116 children, fever (38 to 38,5o C) was present in 80% of them with an average duration of two to four weeks ^30^. Patients tend to have recurrent acute episodes over several months before seeking medical care (table 1). 

In the systematic review of case reports ^11^, the average white blood cell count was 18,000/μl (7,600 - 30,000) and the absolute eosinophil average count was 4,978/μl (0 - 24,000) in 96% of the patients. The results for our cases are presented in table 1.

There is no validated serological test for an accurate diagnosis of angiostrongyliasis, which means that even in cases with high clinical suspicion and diagnostic probability, surgery is indicated ^5^. As informed by Romero-Alegría, *et al.*^11^, diagnosis is done exclusively by pathology in 81% of the cases while in the remaining cases, pathology was accompanied by complementary serological techniques. Currently, confirmatory diagnosis is obtained through surgery and histopathology. The cases reported here were diagnosed through pathological studies (table 1).

The use of anthelmintic drugs such as diethylcarbamazine, thiabendazole, and levamisole is not recommended because it can provoke an erratic migration of the worms or the worsening of the lesions due to the inflammatory response to dead parasites ^31^. Thus, surgery is the treatment of choice ^2^^,^^11^^,^^30^.

Prevention and prophylactic strategies for angiostrongyliasis should be based on education and communication to communities with recognized endemicity. This includes precautions such as:

1. avoiding consumption of snails or animals that can be intermediate or paratenic hosts (particularly snails and slugs);
2. avoiding drinking water from potentially contaminated sources unless an adequate potabilization process is performed;
3. avoiding consuming raw vegetables that have not been properly washed with, ideally, a sodium hypochlorite solution, and
4. controlling the population of rats and snails near houses and in planted fields, especially invasive species such as the giant African snail (*Lissachatina fulica*), which is currently present in most of the Latin American countries, including Colombia ^32^.

## Final remarks

Abdominal angiostrongyliasis is a diagnostic challenge due to multiple reasons. First, its existence is little known. Second, seroprevalence is higher than the number of reported cases, which means that most infections are subclinical and do not require medical attention. Third, underdiagnosis makes it difficult to establish a real incidence. It is important to take into account that due to social and cultural changes during the last decades such as the increase in international tourism and migration trends ^33^, the epidemiological pattern of this disease has changed. For this reason, cases may appear outside of its originally endemic area.

From a public health perspective, the dissemination of *A. costaricensis* and *A. cantonensis* to non-endemic regions and the presence of rats and snails in peridomestic areas represent a substantial risk for future outbreaks. Therefore, it is mandatory to strengthen the projects that aim at raising awareness in the population about the risk of contracting angiostrongyliasis. Moreover, healthcare providers in the American continent should have this parasitic disease in mind to offer timely and adequate medical care.

The geographical distribution of *L. fulica* in Colombia is related to human settlements, deforestation, and industrial activities such as mining, agricultural production, and organic waste disposal ^34^. Therefore, surveillance and control of these intermediate and definitive hosts, as well as health education, should be provided in potential endemic regions to reduce human infection rates.
